# Needle bacterial community structure across the species range of limber pine

**DOI:** 10.1093/ismeco/ycae062

**Published:** 2024-05-09

**Authors:** Dana L Carper, Travis J Lawrence, Dianne Quiroz, Lara M Kueppers, A Carolin Frank

**Affiliations:** Biosciences Division, Oak Ridge National Laboratory, Oak Ridge, TN 37831, United States; Quantitative and Systems Biology Program, University of California, Merced, Merced, CA 95343, United States; Biosciences Division, Oak Ridge National Laboratory, Oak Ridge, TN 37831, United States; Energy & Resources Group, University of California, Berkeley, Berkeley, CA 94720, United States; Energy & Resources Group, University of California, Berkeley, Berkeley, CA 94720, United States; Sierra Nevada Research Institute, University of California, Merced, Merced, CA 95353, United States; Climate and Ecosystem Sciences Division, Lawrence Berkeley National Laboratory, Berkeley, CA 94720, United States; Sierra Nevada Research Institute, University of California, Merced, Merced, CA 95353, United States; Life and Environmental Sciences Department, School of Natural Sciences, University of California, Merced, 5200 Lake Rd, Merced, CA 95343, United States

**Keywords:** microbiome, needle, conifer, phyllosphere, endophytes, bacteria

## Abstract

Bacteria on and inside leaves can influence forest tree health and resilience. The distribution and limits of a tree species’ range can be influenced by various factors, with biological interactions among the most significant. We investigated the processes shaping the bacterial needle community across the species distribution of limber pine, a widespread Western conifer inhabiting a range of extreme habitats. We tested four hypotheses: (i) Needle community structure varies across sites, with site-specific factors more important to microbial assembly than host species selection; (ii) dispersal limitation structures foliar communities across the range of limber pine; (iii) the relative significance of dispersal and selection differs across sites in the tree species range; and (iv) needle age structures bacterial communities. We characterized needle communities from the needle surface and tissue of limber pine and co-occurring conifers across 16 sites in the limber pine distribution. Our findings confirmed that site characteristics shape the assembly of bacterial communities across the host species range and showed that these patterns are not driven by dispersal limitation. Furthermore, the strength of selection by the host varied by site, possibly due to differences in available microbes. Our study, by focusing on trees in their natural setting, reveals real needle bacterial dynamics in forests, which is key to understanding the balance between stochastic and deterministic processes in shaping forest tree-microbe interactions. Such understanding will be necessary to predict or manipulate these interactions to support forest ecosystem productivity or assist plant migration and adaptation in the face of global change.

## Introduction

Plants host diverse communities of above- and belowground microbes that help them tolerate environmental stressors like pathogens, pests, herbivores, drought, and nutrient stress [1, 2]. Greater diversity in bacterial leaf communities has been linked to greater forest tree productivity [3], suggesting an important role for the foliar microbiome in forest tree health. Endophytes, which occur inside leaves, and communities on the leaf surface (the phyllosphere) extend plant phenotypes by producing defense compounds, competing with pathogens for resources, protecting against abiotic stress, producing plant hormones, and fixing atmospheric nitrogen (N) [4–9]. Given the potentially critical contributions of leaf bacterial communities to forest health and ecosystem productivity, it is important to better understand the factors that shape microbial community structure in natural environments.

The relationship between trees and their leaf bacteria depends on a dynamic balance of deterministic and stochastic influences. Deterministic factors, including the host tree’s characteristics and the surrounding abiotic conditions, selectively favor the growth of certain microbes over others. Stochastic events—such as random microbial dispersal and the chance presence or absence of competing or cooperative species—also play a critical role in shaping community structure. The concept of metacommunity, which extends the analysis to interactions across multiple hosts and their environments, offers a framework for dissecting this complexity [10, 11]. In this framework, forest trees can be viewed as patches of microbial habitat that are linked by sets of interacting communities, where dispersal adds community members through bacterial transmission among trees, and where bacterial species are sorted through filtering (ecological selection) by the host tree or the local environment. Though traditional metacommunity theory views patches as embedded in an inhospitable matrix, the environment serves as a reservoir—and hospitable matrix—for many if not most host-associated microbiomes. Leaf microbiomes overlap with soil and air microbiomes [12–14], and the metacommunity of a tree’s microbiome includes not only microbes associated with neighboring plants, but also microbes in the surrounding environment.

Tree foliar bacterial diversity and composition varies across host individuals, host species, space, and time [15, 16] as a result of multiple interacting processes. Selection or filtering by the plant host or environment can be a strong determinant of foliar bacterial communities. For example, trees of different ages select different bacteria [17], and conifers have been found to host some identical bacterial sequence variants in and on needles across space and time, including across continents [7, 17, 18]. Moreover, host species identity and functional traits, such as leaf nitrogen (N), are strong drivers of bacterial community turnover on leaves in both temperate and tropical forest trees [15, 19]. For evergreen trees such conifers, leaf age can also be an important factor in shaping bacterial communities [20], potentially as a consequence of changing characteristics in aging needles [21], or priority effects and accumulation of new microbes over the needle lifespan.

Several studies report a strong signal of host phylogeny on bacterial community composition, likely a result of evolutionary divergence in host selectivity [19, 22]. However, foliar bacteria are typically generalists that can colonize trees as different as gymnosperms and angiosperms, but with stronger selectivity by certain hosts [15, 23]. The ecological and environmental context of a forest tree also shapes its microbiome. Local climate and surrounding tree community have been shown to drive variation in leaf communities among sites [15, 23, 24].

Dispersal can alter the apparent strength of selection effects. Homogenizing dispersal, where high dispersal rates from the surrounding environment such as neighboring trees and other microbial habitats, can overshadow local selection [23, 25]. On the other hand, dispersal limitation could hinder bacteria, including those best fit to the host or climate, from reaching tree foliage. Most bacteria on tree leaves colonize leaves horizontally via dispersal from the environment [26, 27]. Dispersal from neighboring trees is likely an important factor shaping the leaf surface community and colonizing cells from neighbors can overwhelm selection by a host tree, thus altering the degree of specialization between tree hosts and their leaf bacteria [23].

The balance between local selection and dispersal significantly shapes tree leaf microbiomes, with these dynamics varying by geographic location and host phylogeny. Habitat structure influences dispersal rates, affecting microbial communities differently across forests [23]. Laforest-Lapointe et al. [15] highlighted host species’ predominance over site in influencing bacterial communities in Quebec, indicating consistent microbial preferences across locations. Conversely, Finkel et al. [28] reported unique microbial assemblages in Sonoran desert’s Tamarix trees, pointing to dispersal limitations among isolated patches. These contrasts highlight the necessity of studies that unravel the complex interplay between selection and dispersal that shape tree-microbe interactions.

While the factors influencing bacterial communities are increasingly well characterized in controlled environments, parallel research in the complexity of natural forest ecosystems is also important. These natural settings present unique challenges yet offer insights into the real-world dynamics of forest tree leaf communities. Such understanding will be key to predicting and manipulating forest tree-microbe interactions that support forest under accelerated climate change, potentially informing conservation strategies like assisted migration, and projection of tree species’ range shifts. In addition, because of the complexity of such systems, it is important that some studies are large enough to span the variability in ecosystem processes and metacommunity that could shape insights from studies on a smaller spatial scale.

In this study, we investigated the relative importance of selection and dispersal processes in shaping the needle bacterial communities in limber pine (*Pinus flexilis*) and co-ocurring species across the limber pine US geographic range. This widespread Western subalpine conifer has a broad but discontinuous distribution from California to Colorado and New Mexico to Canada. It occupies diverse, often low-fertility environments, potentially aided by partnerships with microbes [7]. We tested three hypotheses regarding the processes structuring bacterial communities of limber pine needles. First, we hypothesized that bacterial community structure would differ across sites and be more strongly influenced by site than selection by the host. This is expected because trees occupying diverse environments experience different stressors, abiotic conditions, and microbial metacommunities. Second, due to the discontinuous nature of subalpine forest in the Western USA, we hypothesized that dispersal limitation would contribute to foliar microbiome composition across the range of limber pine. Third, we hypothesized that the relative importance of dispersal and selection in shaping community structure would vary across sites due to variation in surrounding microbial habitats. Recent findings suggest that high dispersal rates from surrounding trees can swamp selection in the phyllosphere and homogenize communities within a site [23]. Given that the metacommunity structure and dispersal rates from the surrounding habitat are likely to vary across large geographic areas, we expected corresponding variations in the strength of host species identity as a key driver of microbial diversity. Lastly, we hypothesized that needle age structures needle bacterial diversity. Because subalpine conifers retain their leaves for years to decades [29], different needle characteristics between new and old needles as well as accumulation of bacteria over needle lifespan could be important in shaping bacterial diversity. For a comprehensive analysis, we sampled both communities on the needle surface and those associated with needle tissue.

## Materials and methods

**Figure 1:**
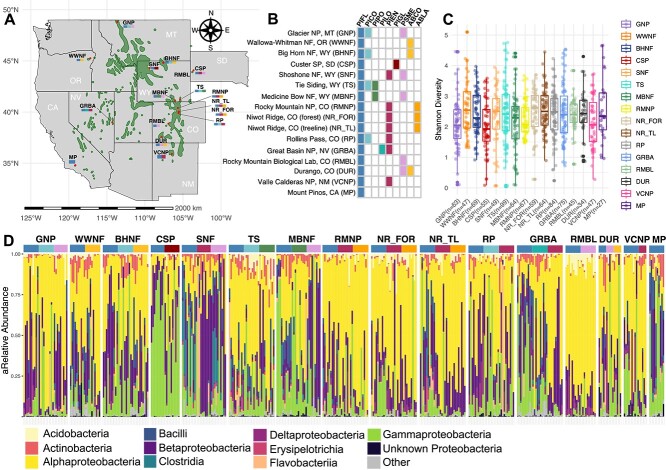
(A, B) Sample sites across the limber pine range, with host species sampled at each site. PIFL: *Pinus flexilis* (limber pine); PICO: *Pinus contorta* (lodgepole pine); PIPO: *Pinus ponderosa* (ponderosa pine); PILO: *Pinus longaeva* (bristlecone pine); PIEN: *Pinus engelmannii* (Engelmann spruce); PIGL: *Picea glauca* (white spruce); PMSE: *Pseudotsuga menziesii* (Douglas fir); ABCO: *Abies concolor* (white fir); ABLA: *Abies lasiocarpa* (subalpine fir). (C) Shannon diversity across sites. (D) Class level taxa bar chart of all surface samples, with old and new needles samples from the same individual merged.

To test our hypotheses, we analyzed the needle communities of limber pine at 16 sites covering the US range of the species (USDA Forest Service, Fire Effects Information System. “*P. flexilis*.”). We examined both surface and tissue communities and included the most dominant 1–2 co-occurring tree species in each site, all belonging to the pine family (see Fig. 1A and B). We sampled the youngest and oldest needle cohorts of *Pinus* species.

### Description of sites

Samples were collected from 16 subalpine sites across the native range of limber pine in August and September 2016 (Fig. 1A and B; Supplementary Table S2). The subalpine forest occupies elevations just below treeline, characterized by low temperatures, high wind, short growing seasons, and thin, coarse textured soil, with low inorganic N availability. Dominant species include pines, spruces, and firs with unique adaptations to the extreme environment such as long individual lifespans and long needle retention time (years to decades). The sites are predominantely surrounded by forest, shrubland, and herbaceous plant communities.

### Sample collection

At each of the 16 sites, 10 limber pine trees were sampled along with 10 trees each of the 1–2 most dominant co-occurring conifer species, if available. For each tree, roughly 10 g of tissue (twig with needles) was cut at breast height from the north side of the tree, using sterile razor blades and placed into sterile bags. Samples were placed on ice and shipped overnight to the University of California, Merced. GPS coordinates and elevation were recorded for each site (Supplementary Table S2) to enable calculation of geographic distances among sites. Climate data variables for each site (Supplementary Tables S3–S6) were extracted from the PRISM interpolated dataset (PRISM Climate Group, Oregon State University, http://prism.oregonstate.edu, created 3 December 2018).

Samples received at the University of California, Merced were stored at 4°C and processed within 3 days of arrival. Twigs from *Pinus* species were subsampled into two age cohorts; the youngest needles, i.e. those that had recently emerged from buds that were most terminal, and the oldest needles, those that were fully developed and the furthest back on the twig.

### Sample processing

From each needle age class, two 1 g samples were placed in 50 mL tubes. To separate tissue and surface communities, we obtained surface microbes via sonication. Samples were immersed in 15 mL of PBS-S buffer and vortexed for 15 seconds, then sonicated for 5 min [30]. The liquid was transferred to new tubes as the surface sample. The needles were sonicated again with fresh PBS-S buffer for 5 min, and the buffer was discarded. Needles were stored at −80°C. Surface samples were centrifuged for 15 min at 3200 g. The supernatant was discarded, and the pellet was resuspended in 1.5 mL of PBS, transferred to a 2 mL tube, and centrifuged for 5 min at 10 000 g. Pellets were frozen until extraction.

### Bacterial enrichment from tissue samples

Using an adjusted protocol for isolating endopshere DNA [31], 1 g sonicated needle samples were divided into two 0.5 g portions and ground into powder in a cryogenic homogenizer (Fisherbrand™ PowerGen™ Cryogenic Homogenizer). Each portion was placed in a 2 mL tube, mixed with 1 mL of potassium phosphate buffer, and combined with 0.3 g of sterile glass beads. After bead beating for 3 min, the tubes were centrifuged at 500 g for 5 min. The supernatants from both portions were combined in a new tube and centrifuged at 12 000 g for 30 min at 4°C. The pellet was mixed with 1 mL of BCE buffer (50 mM Tris–HCl [pH 7.5], 1% Triton X-100, 2 mM mercaptoethanol) and incubated for 30 min. Following another centrifugation at 12 000 g for 30 min at 4°C, the pellet was prepared for DNA extraction. Because we did not sterilize needle samples using ethanol prior to sonication, as was later shown necessary to remove the needle cuticle and the bacteria potentially residing in it [32], we refer to these samples as “tissue” samples rather than endophyte samples.

### DNA extraction, PCR amplification, barcoding, and sequencing

DNA was extracted using the CTAB method as described in Carper et al. [17]. To sequence the 16S rRNA gene and minimize the amplification of plant chloroplast and mitochondrial DNA, a dual-step PCR procedure with chloroplast excluding primers 799f (AACMGGATTAGATACCCKG) and 1115r (AGGGTTGCGCTCGTTG) was employed as described in Carper et al. [17]. All samples, together with 10 controls (without templates), were combined in equimolar amounts in two sequencing pools, with samples from each tree individual divided between the two pools to minimize run effects. Both pools were sent to the University of California, Davis and Berkeley for sequencing on the Illumina MiSeq and HiSeq 2500 platforms, respectively, under a paired-end 250 protocol.

### Read processing

Reads were imported into QIIME2 [33] and demultiplexed. Sequence variants were identified using dada2 [34] within QIIME2 (truncQ = 2, maxN = 0, rm.phix = TRUE, --p-trim-left-f 13, --p-trim-left-r 13, --p-trunc-len-f 250 **\**--p-trunc-len-r 250). Sequences below 250 bp were discarded and variants from multiple runs were combined. Taxonomy was assigned using a classifier trained on the SILVA 118 database [35]. Chloroplast, mitochondria, and sequences from negative controls were excluded. After filtering, all ASVs were assigned to an order, and 99% to family (Supplementary Table S1). Representative sequences were aligned with MAFFT, filtered with MASK in QIIME2, and used to create a phylogenetic tree in RAxML [36]. Samples with fewer than 1000 sequences post-filtering were discarded, along with one sample with only one feature, leaving 948 samples. We then discarded singleton and doubleton ASVs. The sequence variant table, tree, mapping file, and taxonomy were analyzed in phyloseq v. 1.38.0 [37] within R v. 4.1.1 [38].

### Data analysis

To avoid losing data through rarefaction, sequence counts were normalized using Cumulative Sum Scaling (CSS) in the R package MetagenomeSeq v. 1.36.0 [39]. The Shannon diversity index was used to measure alpha diversity. To capture and contrast phylogenetic relationships and species abundance in our analyses, beta diversity was computed using both weighted UniFrac distances and Bray Curtis dissimilarities [40]. We analyzed relationships between bacterial community attributes and site, host species, sample type (surface vs tissue), needle age, and host individual using PERMANOVA [41] with the adonis2 function in vegan. To understand the independent contributions and interactions of variables, we tested the influence of their order in our model. We included individual host ID to account for individual tree effects when multiple samples per tree were analyzed, ensuring independence among observations. In analyses that involved samples from species with and without needle ages (*Pinus* and other species, respectively), young and old needle samples from *Pinus* individuals were consolidated by average OTU abundance. We assessed dispersion homogeneity with betadispr to ensure that detected differences in bacterial community composition were not due to variance within groups, validating the assumptions of our PERMANOVA analysis. Principal Coordinates Analysis (PCoA) was used to illustrate patterns and differences among sites, host species, and needle cohorts, and was conducted in phyloseq.

To investigate the relationship between bacterial communities and geography, we used a Bray Curtis community dissimilarity matrix based on surface samples aggregated by host individual, and a Euclidian environmental distance matrix based on key environmental variables identified from the PRISM data using the bioenv package version 2.6-4 [42]. The bioenv procedure is designed to find the subset of environmental variables that best explain the patterns observed in a community data matrix. Geographic distances were log-transformed following Hayden and Beman [43] and generated using Geographic Distance Matrix Generator [44].

We investigated the distance-decay relationship in limber pine bacterial communities using distance-based Moran’s eigenvector maps, a method that decomposes spatial relationships into orthogonal spatial patterns [45, 46]. For this, we used the PCNM (Principal Coordinates of Neighbor Matrices) function in the spatial package version 0.3.23 [47]. To elucidate the distinct effects of environmental and spatial factors on microbial community structure, we applied distance-based redundancy analysis (db-RDA) with the capscale function in vegan, first with environmental variables, then spatial variables controlled as conditional.

Considering that foliar bacterial communities are influenced by various sources, including local environments that correlate with climate, we partitioned the variation due to climate and land cover on surface limber pine communities (Bray–Curtis distances) with the varpart function in vegan. Land cover data, indicating local environmental makeup, were sourced from a 10 and 25 km radius around each site using FedData v. 3.0.0 [48] from NLCD 2016 [49].

Following data cleaning, we identified a total of 4709 137 sequences, with an average, of 2504 sequences (SD = 8321), and a total of 14 027 ASVs with an average of 44 ASVs per sample (SD = 38). ASVs were detected on average, in 44 samples (SD = 38).

## Results

Like many other plant microbiomes, the needle community of limber pine and co-occurring subalpine conifers was dominated by the phyla Pseudomonadota (85% of all sequences), with the relative proportion of different Pseudomonadota varying across sites (Fig. 1D). At the family level, Acetobacteraceae was the most abundant (making up 36% of the ASVs in samples on average). The most frequent ASV was present in 56% of the samples. Among the top 20 overall most relatively abundant ASVs, 9 belonged to the Acetobacteraceae (Supplementary Table S7). Three of the top 20 ASVs were identical to sequences from endosymbionts of tree-dwelling insect pests (see Supplementary Information).

### Difference between surface and tissue compartments

Both alpha and beta diversity differed significantly between surface and tissue samples.

**Figure 2:**
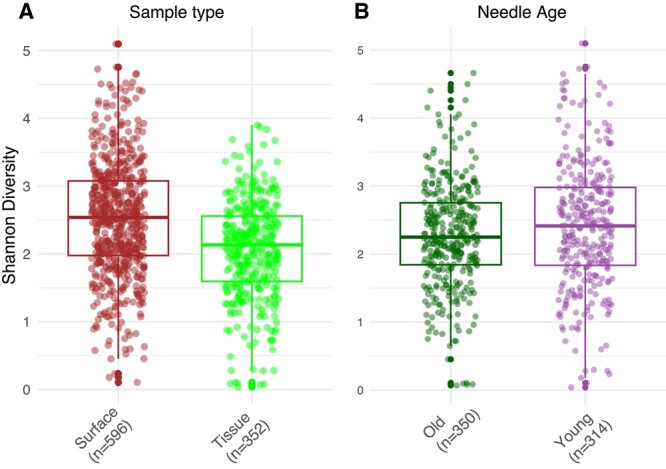
Shannon diversity for (A) sample type, and (B) needle age.

Shannon diversity was significantly higher in surface (mean ± SD: 2.53 ± 0.849, n = 596) than tissue samples (mean ± SD: 2.06 ± 0.728, n = 352; Mann–Whitney U test *P* < 8.048e-16; Fig. 2A). Sample type demonstrated a significant influence on microbial community composition with a high F statistic but accounted for a small proportion of the overall variance (Table 1A). No ASVs were unique to either sample type.

**Table 1:** Results of PERMANOVA analysis for (A) site, host species, sample type, and individual host (HostID) for all samples, with *Pinus* samples merged by age, and (B) for site, needle age, host species, sample type, and individual host for all *Pinus* samples. Variables were incorporated in the order shown here.

			Bray Curtis			Weighted UniFrac		
A: All (*n* = 861)	Variable	df	R2 (%)	F	Pr(>F)	R2 (%)	F	Pr(>F)
	Site	15	17.42	11.97	0.001	14.93	10.12	0.001
	Host species	8	4.77	6.11	0.001	5.29	6.72	0.001
	Sample type	1	1.56	16.04	0.001	1.92	19.60	0.001
	Host ID	371	45.3	1.38	0.001	46.49	1.40	0.001
B: *Pinus* (*n* = 664)	Variable	df	R2 (%)	F	*P*	R2 (%)	F	*P*
	Site	15	17.35	10.80	0.001	15.52	9.93	0.001
	Age	1	0.82	7.67	0.001	2.01	19.83	0.001
	Host species	3	1.70	5.27	0.001	2.23	7.22	0.001
	Sample type	1	1.89	17.61	0.001	2.70	25.95	0.001
	Host ID	205	29.12	1.48	0.001	1.55	1.55	0.001

### Variation in needle community composition and diversity at the limber pine species range scale

An analysis of variance (PERMANOVA) using first all samples followed by only *Pinus* samples (Table 1A and B, respectively) revealed site as a major driver of variation compared with host species identity, sample type (surface vs tissue), or needle age, although these were all statistically significant drivers as well. Individual tree accounted for the highest proportion of variation. PERMANOVA results were similar for the two distance metrics used, and the order in which variables were incorporated into our model did not influence their contribution to variation in community composition.

Alpha (Shannon) diversity varied significantly among sites (Kruskal–Wallis, *P*-value = 9.35e-05; Fig. 1C). Young needles (mean ± SD: 2.27 ± 0.780, n = 350) had significantly higher diversity than old needles (mean ± SD: 2.42 ± 0.934, n = 314); Mann–Whitney U test *P* < 1.204e-13 (Fig. 2B). Communities from different host species did not differ significantly in diversity at this scale.

**Figure 3:**
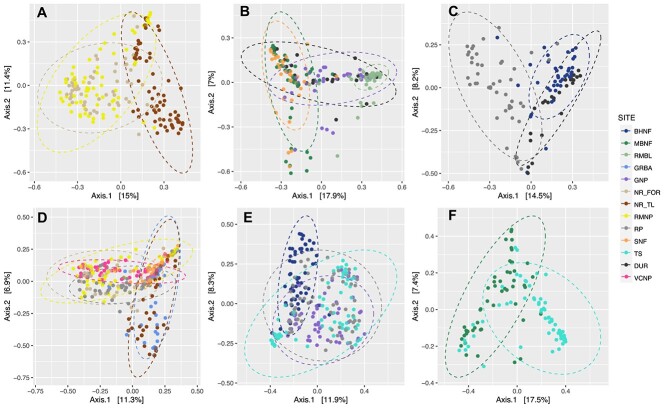
PCoA of Bray–Curtis distances for host species and sites. (A) Host species PIFL, PIEN, and ABLA at sites NR_TL, NR_FOR, and RMNP, (B) host species PIFL, PSME at sites SNF, DUR, RMBL, MBNF, GNP, (C) host species PIFL and ABCO at sites DUR, BHNF, and WWNF, (D) host species PIFL and PIEN at sites GRBA, VCNP, RP, NR_FOR, NR_TL, RMNP, and SNF, (E) host species PIFL and PICO at sites RP, GNP, BHNF, and TS, (F) host species PIFL and PIPO at sites MBNF and TS.

**Figure 4:**
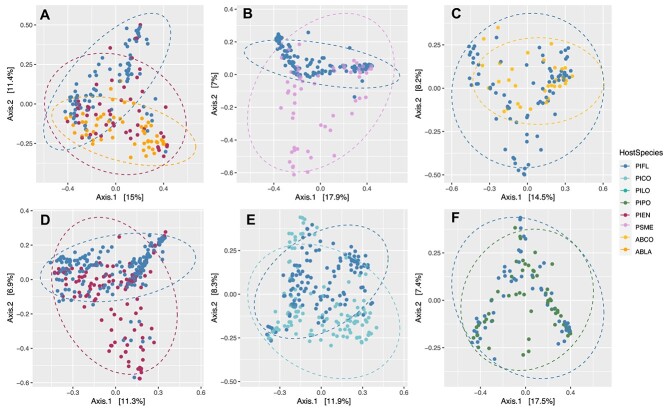
PCoA of Bray–Curtis distances for host species and sites. (A) Host species PIFL, PIEN, and ABLA at sites NR_TL, NR_FOR, and RMNP, (B) host species PIFL, PSME at sites SNF, DUR, RMBL, MBNF, GNP, (C) host species PIFL and ABCO at sites DUR, BHNF, and WWNF, (D) host species PIFL and PIEN at sites GRBA, VCNP, RP, NR_FOR, NR_TL, RMNP, and SNF, (E) host species PIFL and PICO at sites RP, GNP, BHNF, and TS, (F) host species PIFL and PIPO at sites MBNF and TS.

To further evaluate the impact of site and host species identity on the needle bacterial communities, we examined the six data subsets (surface samples only, young and old *Pinus* samples merged by age) where the same species of trees were sampled across multiple sites. For example, subalpine fir and Engelmann spruce were sampled alongside limber pine in Rocky Mountain National Park and at both forest and treeline at Niwot Ridge (Table 2; see also Fig. 1B). In five of six comparisons, site predominantly influenced community variation, and for the majority of data subsets, Bray–Curtis distances resulted in higher R2 for site (Table 2, Figs 3 and 4).

**Table 2:** Results for PERMANOVA analysis for site and host species data subsets where the same species of trees were sampled across multiple sites. Variables were incorporated in the order shown here. Only surface samples were used, and *Pinus* samples from different needle age cohorts were merged.

				Bray Curtis			Weighted Unifrac		
Sites	Species	Var.	Df	R2 (%)	F	p	R2 (%)	F	p
NR_TL, NR_FOR, RMNP	PIFL, PIEN, ABLA	Site	2	18.37	10.92	0.001	7.52	3.82	0.002
		Species	2	10.14	6.03	0.001	8.68	4.40	0.001
SNF, DUR, RMBL, MBNF, GNP	PIFL, PSME	Site	4	25.30	7.31	0.001	28.96	8.88	0.001
		Species	1	3.78	4.37	0.001	4.18	5.12	0.003
DUR, BHNF, WWNF	PIFL, ABCO	Site	2	22.40	6.96	0.001	8.97	2.33	0.017
		Species	1	3.60	2.24	0.004	2.53	1.32	0.227
GRBA, VCNP, RP, NR_FOR, NR_TL, RMNP, SNF	PIFL, PIEN	Site	6	22.81	6.53	0.001	17.41	4.65	0.001
		Species	1	3.87	6.66	0.001	3.99	6.40	0.001
RP, GNP, BHNF, TS	PIFL, PICO	Site	3	16.99	5.50	0.001	16.03	5.14	0.001
		Species	1	5.71	5.54	0.001	6.06	5.84	0.001
MBNF, TS	PIFL, PIPO	Site	1	17.79	8.79	0.001	21.91	10.90	0.001
		Species	1	7.33	3.62	0.001	3.71	1.85	0.097

Site-specific tree microbiomes might result from dispersal limitations causing distance-decay, or reduced community similarity with increased geographic distance [28]. We did not find a distance-decay relationship in limber pine samples. Partial db-RDA revealed an effect of environmental variables on microbial community composition variance. Controlling for key environmental variables identified with bioenv (Table 3), spatial patterns were nonsignificant, whereas environmental variables, when controlling for spatial influences, accounted for 19.3% of the variance (*P* < 0.001).

**Table 3:** The set of environmental variables identified using bioenv that best match the Bray Curtis distances. Bionev provides a single correlation coefficient (Spearman’s rank correlation) for the subset of selected variables.

Best parameters	Correlation
Vapor pressure maximum (30 year normal)	0.112
Precipitation (summer)	
Dew point temperature (summer)	
Vapor pressure minimum (summer)	

**Figure 5:**
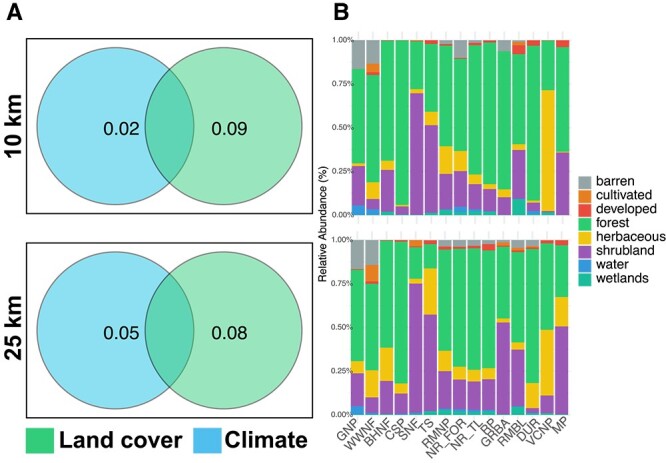
A) Variation partitioning of bacterial community composition (proportion of variance explained) as a function of land cover (25 and 10 km radius) and climate. B) Relative abundance of land cover categories across sites.

Next, we explored whether climate’s effect on limber pine needle community variation was instead, or in addition, attributed to differences in surrounding habitat. Previous studies suggest that neighboring forests (25 km radius) greatly impact tree foliar communities [23]. Given the correlation between forest structure and climate, apparent climate effects might stem from variations in microbial sources from neighboring vegetation and land cover types. We utilized land cover data at a 10 and 25 km radius, denoting area-specific land types, to discern the influence of climate versus habitat structure on bacterial communities. Our 16 subalpine sites were primarily surrounded by forest, shrubland, herbaceous, and barren land, with minimal developed or agricultural areas (Fig. 5B). We assessed the bacterial community variation due to climate and land cover type abundance. Results showed both factors significantly influenced bacterial communities, but land cover type had a greater impact than climate (Fig. 5A).

### Variation in needle community composition and diversity at the site scale

**Figure 6:**
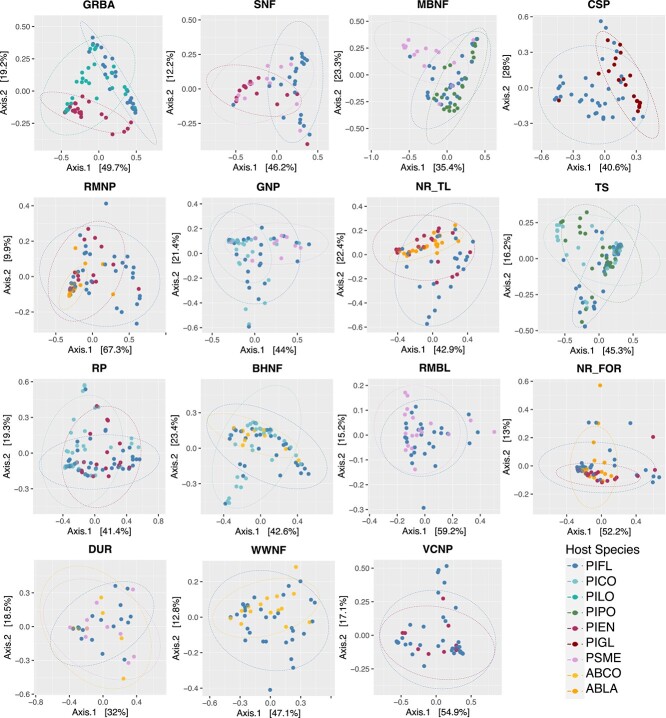
PCoA of weighted UniFrac distances for each site.

For sites with multiple tree species sampled (excluding Mount Pinos, CA), we examined community structure differences between host species for surface samples, with *Pinus* samples merged by age (Table 4, Fig. 6). The variation explained by host species ranged from 31% at Great Basin National Park, NV (using Weighted Unifrac) to being nonsignificant with both distance metrics at Durango, CO. Weighted UniFrac yielded higher R2 values than Bray–Curtis in several sites (e.g. CSP, GRBA, MBNF, SNF), indicating a trend toward stronger effect of host species on microbial community composition with a phylogenetic metric. However, the pattern was not consistent across all sites. Within our sites, alpha diversity was mostly not significantly different across tree species, with the exception of GNP, CSP, and RMNP (Supplementary Table S10, Fig. S2).

**Table 4:** Results of PERMANOVA analysis for host species within each site. Only surface samples were used, and *Pinus* samples from different needle age cohorts were merged.

			**Bray Curtis**			**Weighted Unifrac**		
**Site**	**Species**	**df**	**R2 (%)**	**F**	**Pr(>F)**	**R2 (%)**	**F**	**Pr(>F)**
BHNF	ABCO PIFL PICO	2	19.12	3.19	0.001	13.62	2.13	0.04
CSP	PIFL PIGL	1	18.17	3.78	0.001	26.14	6.02	0.001
DUR	ABCO PIFL PSME	2	14.63	1.03	0.38	7.12	0.46	0.93
GNP	PIFL PSME PICO	2	14.72	2.24	0.001	17.25	2.71	0.02
GRBA	PIEN PIFL PILO	2	18.88	3.14	0.001	31.34	6.16	0.001
MBNF	PIFL PSME PIPO	2	21.40	3.54	0.001	26.89	4.78	0.001
NR_FOR	ABLA PIEN PIFL	2	16.36	2.64	0.001	7.68	1.12	0.33
NR_TL	ABLA PIEN PIFL	2	21.65	3.73	0.001	16.68	2.70	0.01
RMBL	PIFL PSME	1	13.04	2.70	0.01	9.28	1.84	0.11
RMNP	ABLA PIEN PIFL	2	21.97	3.80	0.001	24.50	4.38	0.003
RP	PIEN PIFL PICO	2	14.64	2.31	0.001	10.99	1.67	0.07
SNF	PIEN PIFL PSME	2	21.67	3.60	0.001	31.18	5.89	0.001
VCNP	PIEN PIFL	1	17.27	2.71	0.001	16.14	2.50	0.07
WWNF	ABCO PIFL	1	13.75	2.87	0.002	8.35	1.64	0.17
TS	PIFL PICO PIPO	2	22.15	3.84	0.001	16.89	2.74	0.003

**Figure 7:**
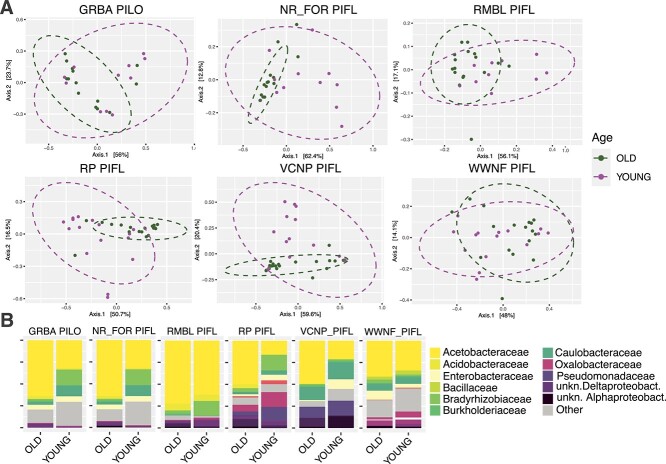
(A) PCoA of weighted Unifrac distances for needle age for species site combinations that were significant in the PERMANOVA analysis (Table 5). (B) Relative abundances of bacterial families in young and old needles for the same species/site combinations as in A.

Next, we assessed the impact of needle age on community turnover for each pine host species and site (Table 5). Individual host was again the largest driver of variation. While needle age did not significantly affect most species in most sites, it strongly influenced community variation in some, like limber pine at Rollins Pass, Niwot Ridge, (forest) and Rocky Mountain National Laboratory, all in CO, where needle age explained up to 25% of the variation (Table 5; Fig. 7A). Older needles showed less compositional variation and a higher presence of Acetobacteraceae (Fig. 7B). The phylogenetic distance metric (weighted Unifrac) consistently captured more variation due to needle age than the compositional metric (Table 5). No alpha diversity difference was observed between young and old needles (data not shown).

**Table 5:** Results of PERMANOVA analysis for needle age for each *Pinus* species / site combination. Rows where results were significant using one or both metrics are shaded. Only surface samples were used, and HostID was included in the model but omitted here for space.

**Site**	**Species**	**Df**	**R bray (%)**	**F bray**	**p bray**	**R wuni (%)**	**F wuni**	**p wuni**
BHNF	PIFL	1	8.08	1.84	0.034	9.02	3.62	0.035
BHNF	PICO	1	2.84	0.72	0.749	3.78	1.31	0.251
CSP	PIFL	1	2.60	1.20	0.273	5.61	1.37	0.294
DUR	PIFL	1	10.06	0.89	0.561	9.32	1.19	0.334
GNP	PIFL	1	6.56	1.42	0.086	4.02	0.88	0.507
GNP	PICO	1	6.33	1.29	0.161	6.49	1.35	0.224
GRBA	PIFL	1	4.42	0.76	0.793	6.07	0.95	0.373
GRBA	PILO	1	6.41	1.19	0.240	12.91	2.69	0.044
MBNF	PIFL	1	6.80	1.13	0.286	6.01	1.11	0.388
MBNF	PIPO	1	6.19	1.37	0.166	4.55	0.99	0.374
MP	PIFL	1	4.72	0.78	0.893	3.51	0.88	0.471
NR_FOR	PIFL	1	11.16	2.29	0.014	17.75	3.45	0.038
NR_TL	PIFL	1	5.12	1.03	0.408	5.61	1.04	0.393
RMBL	PIFL	1	12.92	3.37	0.001	22.30	6.22	0.005
RMNP	PIFL	1	8.38	1.92	0.028	9.31	1.93	0.128
RP	PIFL	1	14.17	3.41	0.002	25.00	6.39	0.006
RP	PICO	1	8.02	1.79	0.023	11.72	2.47	0.062
SNF	PIFL	1	5.44	1.29	0.218	4.88	1.40	0.214
TS	PIFL	1	9.39	1.67	0.043	9.78	1.58	0.218
TS	PICO	1	5.13	1.06	0.396	3.81	0.83	0.572
TS	PIPO	1	8.58	2.07	0.028	6.30	2.00	0.129
VCNP	PIFL	1	14.84	3.64	0.002	21.20	6.01	0.003
WWNF	PIFL	1	7.65	1.52	0.088	15.62	3.67	0.019

## Discussion

Our study represents one of the most comprehensive examinations of the needle microbiome to date, analyzing both surface tissue communities across 9 host species and 16 sites, spanning a latitudinal gradient of 1430 km. From these needle samples throughout the limber pine range, we found that local site factors exert a greater influence on bacterial community assembly than host species identity. Even though many bacterial taxa remained consistent across the range, each forest had a distinct microbiome profile. This contrasts with previous research where host species was the main factor for tree foliar communities on a smaller spatial scale but including both conifers and deciduous species [15, 50]. Our results underscore the significance of host phylogeny and geographic scale in microbiome studies. We also found that selection’s influence on community structure, driven by host identity and needle age, varied across sites.

Surface communities showed greater diversity than those from tissue (Fig. 2A), but sample type explained only a fraction of the variation in the bacterial communities. We did not clean needles with ethanol after removing surface microbes [32], potentially leaving some needle cuticle associated microbes in our tissue samples.

The influence of host species on bacterial communities varied across sites. Though we sampled diverse tree species co-existing with limber pine, the effect did not increase with greater host phylogenetic differences. For instance, sites with a broader phylogenetic range, like those including *Pseudotzuga* or *Abies* (e.g. in Big Horn National Forest, WY, Wallowa-Whitman National Forest, OR), did not show stronger effects than sites with just *Pinus* and *Picea* species (e.g. Great Basin National Park, NV). It is also important to point out that most of the variation was attributed to individual host tree (Table 1), which is expected given our small amount of sample, but also underscores that most factors driving variation in needle communities remain unknown, and that the communities are likely influenced by stochastic factors.

Within most locations, the age of needle cohorts did not markedly influence bacterial communities. However, in select site-species combinations, it played a substantial role. In these instances, older needles showed less bacterial variation and an enrichment in Acetobacteraceae taxa, hinting at deterministic influences. A potential explanation is that older needles at certain sites may have been exposed to herbivore attacks, whereas those in other locations remained unaffected. In addition, pine needle longevity varies with site [51, 52], and our categories (young and old) are broad and may have resulted in needles of different ages being sampled in different sites. Needle community shifts with age could be the result of ongoing accumulation of bacteria through leaf lifespan. However, there was no significant difference in alpha diversity between young and old needle communities. Alternatively, late successional leaf surface microbial communities can become relatively stable against invasion [53], and the community may not change much after it is first established. If this is the case for subalpine needle bacterial communities, the difference in young and old needle communities may simply be the consequence of different metacommunity dynamics at the time of needle growth, resulting in different stable microbial communities.

Interestingly, the magnitude of R2 values in PERMANOVA analysis differed between the phylogenetic and compositional metrics, varying by scale and driver. At broader geographic scales involving multiple sites, a trend emerged where the compositional Bray–Curtis metric resulted in higher R2 values for the variable “site”, suggesting that physical and biotic environmental factors are major determinants of microbial diversity across sites. Conversely, at a more localized scale, Weighted UniFrac provided higher R2 values for host species and needle age, indicating that these host-related factors are predominantly influenced by phylogenetic relationships. This suggests that microbial communities may converge toward or diverge from certain phylogenetic lineages as needles age and across different host species, reflecting ecological dynamics such as niche specialization or competitive exclusion.

The needle community structure at a site likely reflects the interplay of dispersal, selection, and drift. However, our findings did not support the hypothesis that dispersal limitation drive subalpine conifer leaf microbiome differences. We did not observe a pattern where geographically closer sites had similar microbiomes. For instance, needle communities at Niwot Ridge’s treeline and subalpine forest, despite their proximity, differed more from each other than from distant sites (Fig. 3 and Supplementary Fig. S1). Dominant ASVs, primarily from the Acetobacteraceae, were consistent across limber pine’s range (Supplementary Tables S7 and S8). Potential dormancy in a significant portion of the needle-associated community might obscure the expected distance-decay relationship [54].

Leaf microbiomes are transmitted through aerosols, soil, insects, pollen, and other plant parts [26]. Acetobacteraceae, prevalent in air samples, especially high-elevation and forest sites [55–58], dominated our data across the limber pine range, indicating a shared metacommunity from across the large geographic region sampled here. This dominance suggests Acetobacteraceae’s high dispersal and adaptability, including tolerance to cold and UV radiation. These bacteria, part of the Rhodospirillales order, are prominent in high-altitude air samples [57]. Many of the dominant ASVs are identical to or closely related to bacteria isolated from cold environments such as high elevation air or glacier surfaces (data not shown). Interestingly, an aerobiome study showed Acetobacteraceae ASV spikes in forests after rain [58], hinting at rain’s role in releasing tree surface bacteria. While rain may minimally impact leaf communities [59, 60], it can produce bioaerosols [61], potentially influencing tree-to-tree bacterial transmission.

Local factors had a strong impact on needle community structure across the geographic range of limber pine. Because site-to-site differences were not caused by limited dispersal, it should instead reflect a combination of local environmental filtering (by local climate and host trees) and the local species pool (i.e. the community of microbes available to colonize from surrounding trees, air, soil, insects, and other plants). Climate had a modest influence on the bacterial communities of limber pine and neighboring conifers, with the surrounding land cover composition (forest and other types) at 10 and 25 km distances, explaining slightly more, suggesting that land cover shapes the abundance and identity of bacteria available to colonize needles.

The sampled sites might have been exposed to varying natural and human-made disturbances, potentially influencing tree microbiomes. For instance, urban intensification can decrease Alphaproteobacteria abundance [50], a group whose abundance fluctuated across our sites. Periodic events like fires, droughts, and insect outbreaks in subalpine forests can alter the aerobiome composition, especially wildfire smoke [62], impacting the phyllosphere microbiome.

Differences in dispersal rates or metacommunity structure could also explain why host species identity and needle age are strong drivers of community variation in some sites, and weak or nonexistent in others. Dispersal can affect beta diversity in different ways; it can homogenize communities [63, 64], but it can also increase differences in species composition due to stronger priority effects under increased dispersal rates, as demonstrated by manipulation of dispersal of microbes in nectar via pollinators [65]. For differences in leaf communities among tree species, there is evidence that increased dispersal has a homogenizing effect. Selection of microbial taxa on *Acer saccharum* (sugar maple) leaf surface in temperate and boreal forests can be outweighed by high dispersal from neighboring trees at a 25 km radius [23]. Similar findings at a smaller scale have been reported in experimental tomato, pepper, and bean plants, where the outcome of plant selection in the phyllosphere was shaped by the identity and local biomass of neighboring plants [25]. Moreover, similar dynamics are at play in more distantly related hosts systems such as the zebrafish, where dispersal can overwhelm host selection in the gut microbiome [66]. Thus, it is possible that the strong effect of host species in some sites like Great Basin NP, Shoshone NF, and Custer SP reflects lower dispersal rates, while weaker effects at other sites reflect higher dispersal rates. However, dispersal does not always lead to homogenization of foliar communities; fungal communities in vineyards and nearby forest patches have been found to increasingly differentiate rather than homogenize over the growing season [67].

Alternatively, or in addition, the strength of host species filtering could vary with site if needle chemistry varies by site or trees are or have experienced site-specific stressors like drought and pathogen or insect infestation. For example, variation in needle terpene profiles, which could potentially shape phyllosphere communities, has been shown to correlate with historical herbivore attack [68].

## Conclusion

Our comprehensive study of the needle microbiome across the limber pine range provides new insights into the factors shaping microbial communities in forest ecosystems. We found that local site characteristics, rather than host species identity or dispersal limitations, predominantly influence the assembly of bacterial communities. This suggests that environmental filtering by site-specific factors plays a more crucial role in shaping these communities than previously understood.

Our results also demonstrate that the balance between deterministic factors like environmental filtering and stochastic events varies significantly across large geographic areas. This variability contributes to the complex dynamics of microbial community assembly in natural ecosystems, highlighting that microbial diversity and composition are influenced by a mosaic of ecological processes.

By focusing on the ecological and evolutionary interactions within these communities, future research could further elucidate how microbial partners contribute to the adaptive capacity of forest trees. This could be particularly valuable for conservation strategies and enhancing forest resilience in the face of climate variability.

## Supplementary Material

Supplementary_Information_revised_ycae062

## Data Availability

Raw sequence reads and associated metadata were deposited in the NCBI SRA database under bioproject PRJNA497738: https://www.ncbi.nlm.nih.gov/bioproject/PRJNA497738.
