# Supplementary material for: Needle bacterial community structure across the species range of limber pine
Source: ISME Commun. 2024 May 9;4(1):ycae062. doi: 10.1093/ismeco/ycae062 (PMC11128189; doi:10.1093/ismeco/ycae062)
Supplement: Supplementary_Information_revised_ycae062 [file supplementary_information_revised_ycae062.docx]

Supplementary Supporting Information

**Title: Needle bacterial community structure across the species range of limber pine**

**Running title: Limber pine foliar microbiome**

**Authors:** Dana L. Carper, Travis J. Lawrence, Dianne Quiroz, Lara M. Kueppers, A. Carolin Frank

Three of the top 20 ASVs were identical to sequences from endosymbionts of tree-dwelling insect pests. One sequence variant was identical to Ca. *Annandia pinicola*, an endosymbiont of the sap-feeding pine woolly aphid (*Pineus coloradensis*) (von Dohlen et al., 2017). Another ASV was identical to Ca. *Vallotia cooleya*, an endosymbiont of the gall adelgid (*Adelges cooleyi*) (von Dohlen et al., 2017), which has a two year life cycle alternating between spruces and Douglas fir (Havill & Foottit, 2007). A third ASV was identical to strains of Ca. *Uzinura diaspidicola*, an endosymbiont of the pine needle scale insect *Chionaspis pinifoliae* (Gruwell, Flarhety, & Dittmar, 2012), a common pest on pine and spruce in North America (Shour, M.H., 1987). Despite their high relative abundance in some samples, insect endosymbiont ASVs were not detected as core ASVs in any site (i.e. never occurred in more than 50% of samples in a site).

The effect of insect infestation at some sites was apparent in our data, with ASVs matching the obligate, vertically transmitted insect endosymbionts Candidatus *Annandia*, and Candidatus *Vallotia cooleyia* present at high relative abundance in some site/species combinations. The presence of these sequences indicates that our protocol for removing surface microbes may have been insufficient. These endosymbionts are not likely to survive without their host and likely originated from eggs stuck in the needle wax. The insect hosts of these endosymbionts, *P. coloradensis* and *A. cooleyi* lay their eggs in wax-wool, scales, or waxy threads that stick to the needle surface (Havill & Foottit, 2007; Shour, M.H., 1987). Although insect endosymbionts are unlikely to be true members of the needle microbiome, we decided to keep the sequences in the dataset for two reasons: First, removal of the three insect endosymbiont ASVs did not affect the results (data not shown), and second, they are unlikely to represent the only inactive members of the needle microbiome, and we lack criteria for identifying other inactive members. In addition, insect endosymbiont ASVs in the plant microbiome may be useful as indicators for insect pest outbreaks.

von Dohlen, C. D., Spaulding, U., Patch, K. B., Weglarz, K. M., Foottit, R. G., Havill, N. P., & Burke, G. R. (2017). Dynamic Acquisition and Loss of Dual-Obligate Symbionts in the Plant-Sap-Feeding Adelgidae (Hemiptera: Sternorrhyncha: Aphidoidea). *Frontiers in Microbiology*, *8*, 1037.

Gruwell, M. E., Flarhety, M., & Dittmar, K. (2012). Distribution of the Primary Endosymbiont (Candidatus Uzinura Diaspidicola) Within Host Insects from the Scale Insect Family Diaspididae. *Insects*, *3*(1), 262–269.

Shour, M.H. (1987). Host range and geographic distribution of Chionaspis heterophyllae Cooley and C. pinifoliae (Fitch) (Homoptera: Diaspididae). *Proceedings of the Indiana Academy of Sciences*, *96*, 297–304.

Havill, N. P., & Foottit, R. G. (2007). Biology and Evolution of Adelgidae. *Annual Review of Entomology*, *52*(1), 325–349.

**Supplementary tables and figures**

Supplementary Table 1: Successful taxonomy assignment per taxa level

| Taxonomic Level | Percentage assigned |
| --- | --- |
| Phylum | 100 |
| Class | 100 |
| Order | 99.8 |
| Family | 99.0 |
| Genus | 55 |
| Species | 24 |

Supplementary Table 2: Collection sites and dates

| Site | Latitude | Longitude | Elevation (m) | Date Collected |
| --- | --- | --- | --- | --- |
| Mt. Pinos, CA | 34.81 | -119.14 | 2700 | 8/8/16 |
| Great Basin National Park, NV | 39.00 | -114.30 | 3153 | 9/26/16 |
| Valles Caldera National Park, NM | 35.91 | -106.48 | 2642 | 8/13/16 |
| Durango, CO | 37.57 | -107.81 | 2585 | 8/14/16 |
| Rocky Mountain Biological Laboratory, CO | 38.87 | -106.93 | 2870 | 9/14/16 |
| Rollins Pass, CO | 39.90 | -105.59 | 2853 | 9/9/16 |
| Niwot Ridge (Forest Site), CO | 40.05 | -105.58 | 3060 | 9/5/16 |
| Niwot Ridge (Treeline Site), CO | 40.04 | -105.54 | 3430 | 9/5/16 |
| Rocky Mountain National Park, CO | 40.40 | -105.66 | 3361 | 9/12/16 |
| Medicine-Bow National Forest, WY | 41.14 | -106.04 | 2452 | 8/17/16 |
| Tie Siding, WY | 41.22 | -105.39 | 2586 | 8/16/16 |
| Shoshone National Forest, WY | 44.31 | -109.28 | 2496 | 8/25/16 |
| Big Horn National Forest, WY | 44.81 | -107.61 | 2598 | 8/23/16 |
| Custer State Park, SD | 44.08 | -103.62 | 1457 | 8/28/16 |
| Glacier National Park, MT | 48.80 | -113.64 | 1500 | 8/30/16 |
| Wallowa-Whitman National Forest, OR | 45.26 | -117.18 | 2444 | 8/31/16 |

Supplementary Table 3: PRISM data of minimum and mean temperature for each site

|  |  | Precipitation (inches) | | | | |
| --- | --- | --- | --- | --- | --- | --- |
| Site | Days since last precipitation | 30 year normal | 2016 | Month sampled | Day sampled | Day before sampling |
| Mt. Pinos, CA | 93 | 0.21 | 14.22 | 0 | 0 | 0 |
| Great Basin National Park, NV | 1 | 1.48 | 25.19 | 0.75 | 0.04 | 0 |
| Valles Calderas National Preserve, NM | 0 | 4.69 | 20.28 | 4.51 | 0 | 0.02 |
| Durango, CO | 5 | 2.95 | 31.19 | 5.5 | 0 | 0 |
| Rocky Mountain Biological Laboratory, CO | 0 | 2.19 | 25.47 | 1.2 | 0.06 | 0 |
| Rollins Pass, CO | 7 | 1.8 | 27.16 | 0.86 | 0 | 0 |
| Niwot Ridge (Forest Site), CO | 2 | 1.89 | 30.83 | 0.67 | 0 | 0.17 |
| Niwot Ridge (Treeline Site), CO | 2 | 2.28 | 38.05 | 0.67 | 0 | 0.12 |
| Rocky Mountain National Park, CO | 0 | 2.04 | 29.73 | 0.64 | 0 | 0 |
| Medicine-Bow National Forest, WY | 12 | 1.28 | 12.72 | 0.49 | 0 | 0 |
| Tie Siding, WY | 1 | 1.21 | 20.53 | 0.96 | 0.43 | 0 |
| Shoshone National Forest, WY | 11 | 1.86 | 19.67 | 0.77 | 0 | 0 |
| Big Horn National Forest, WY | 3 | 1.15 | 25.31 | 1.1 | 0 | 0 |
| Custer State Park, SD | 0 | 2.14 | 16.82 | 3 | 0 | 0.15 |
| Glacier National Park, MT | 3 | 1.9 | 53.04 | 3.05 | 0 | 0.07 |
| Wallowa-Whitman National Forest, OR | 12 | 1.3 | 44.69 | 0.5 | 0 | 0 |

Supplementary Table 4: PRISM data of minimum and mean temperature for each site

|  | Temperature minimum (Degrees F) | | | | | Temperature mean (Degrees F) | | | | |
| --- | --- | --- | --- | --- | --- | --- | --- | --- | --- | --- |
| Site | 30 year normal | 2016 | Month sampled | Day sampled | Day before sampling | 30 year normal | 2016 | Month sampled | Day sampled | Day before sampling |
| Mt. Pinos, CA | 51.7 | 38.4 | 56.2 | 54.6 | 54 | 62.3 | 49.9 | 67.8 | 66 | 65.9 |
| Great Basin National Park, NV | 35.6 | 27.9 | 36.9 | 29.6 | 27.5 | 47.7 | 37.9 | 47.8 | 40.3 | 37.6 |
| Valles Calderas National Park, NM | 42 | 27.1 | 41.4 | 45.6 | 46.3 | 56.6 | 41.2 | 54.6 | 60.8 | 59.8 |
| Durango, CO | 44.7 | 30.9 | 44.7 | 42.3 | 42.3 | 59.5 | 42.1 | 55.3 | 55.7 | 55.8 |
| Rocky Mountain Biological Laboratory, CO | 36.5 | 24.3 | 34.9 | 37.4 | 35.5 | 49.3 | 37.2 | 48.9 | 48.2 | 50 |
| Rollins Pass, CO | 35.5 | 31.1 | 41 | 44.3 | 45.1 | 50 | 41.5 | 53.2 | 57.5 | 56.9 |
| Niwot Ridge (Forest Site), CO | 34.7 | 27.9 | 37 | 40.1 | 43.5 | 47.4 | 38.2 | 49.3 | 52 | 55.3 |
| Niwot Ridge (Treeline Site), CO | 30.8 | 25.2 | 35 | 37.6 | 40.5 | 42.4 | 35.4 | 46.6 | 48.7 | 51.7 |
| Rocky Mountain National Park, CO | 31.9 | 27.6 | 36.7 | 46.4 | 29.7 | 43.9 | 38.2 | 49.7 | 58.2 | 48.8 |
| Medicine-Bow National Forest, WY | 43.8 | 27.2 | 40 | 38.9 | 38 | 59.3 | 40.6 | 57.1 | 57.5 | 56.9 |
| Tie Siding, WY | 42.2 | 31.8 | 47.3 | 35.1 | 38.6 | 56.7 | 40.5 | 57.5 | 44.2 | 51.8 |
| Shoshone National Forest, WY | 44.9 | 32.1 | 45.6 | 43.3 | 45.7 | 58.6 | 42.6 | 59.5 | 60.3 | 61.7 |
| Big Horn National Forest, WY | 44.9 | 29.4 | 45.1 | 44.4 | 45.8 | 56.2 | 38.9 | 56.3 | 58.6 | 59.3 |
| Custer State Park, SD | 48.6 | 31.3 | 47.6 | 45 | 42.8 | 64.3 | 44.9 | 62.5 | 60.8 | 54.3 |
| Glacier National Park, MT | 46.1 | 33.4 | 45.8 | 42.1 | 44.3 | 58.5 | 41.7 | 58.1 | 61.4 | 54.6 |
| Wallowa-Whitman National Forest, OR | 42.5 | 29.3 | 42.3 | 48.1 | 53.7 | 54.1 | 37.5 | 53.4 | 58.6 | 61.5 |

Supplementary Table 5: PRISM data of maximum and dew point temperature for each site

|  | Temperature maximum (Degrees F) | | | | | Dew point temperature (Degrees F) | | | | |
| --- | --- | --- | --- | --- | --- | --- | --- | --- | --- | --- |
| Site | 30 year normal | 2016 | Month sampled | Day sampled | Day before sampling | 30 year normal | 2016 | Month sampled | Day sampled | Day before sampling |
| Mt. Pinos, CA | 72.8 | 61.4 | 79.4 | 77.4 | 77.7 | 34.3 | 23.4 | 27.2 | 24.8 | 28.4 |
| Great Basin National Park, NV | 59.7 | 47.8 | 58.6 | 51 | 47.6 | 17.7 | 14.3 | 19.3 | 16.9 | 16.4 |
| Valles Calderas National Park, NM | 71.2 | 55.3 | 67.9 | 76 | 73.3 | 42.9 | 21.1 | 41.9 | 36.9 | 40.6 |
| Durango, CO | 74.3 | 53.3 | 65.9 | 69.1 | 69.3 | 40.5 | 21.5 | 38.3 | 28.4 | 24.6 |
| Rocky Mountain Biological Laboratory, CO | 62.1 | 50.2 | 63 | 59 | 64.5 | 29.6 | 18.2 | 26.9 | 33.6 | 31.1 |
| Rollins Pass, CO | 64.5 | 51.9 | 65.5 | 70.7 | 68.7 | 31.5 | 20 | 28.4 | 19.2 | 23.4 |
| Niwot Ridge (Forest Site), CO | 60.1 | 48.5 | 61.6 | 63.8 | 67.1 | 29.6 | 18.5 | 26.6 | 32.3 | 37.6 |
| Niwot Ridge (Treeline Site), CO | 54 | 45.5 | 58.1 | 59.9 | 62.9 | 26 | 16.1 | 24.4 | 29.8 | 35.7 |
| Rocky Mountain National Park, CO | 55.9 | 48.9 | 62.8 | 70 | 67.9 | 29.3 | 20 | 27.2 | 18.7 | 13 |
| Medicine-Bow National Forest, WY | 74.9 | 54 | 74.1 | 76.1 | 75.7 | 40.4 | 22.4 | 34.9 | 33.8 | 31.3 |
| Tie Siding, WY | 71.2 | 49.2 | 67.7 | 53.2 | 65 | 32.7 | 17.5 | 26.3 | 31.3 | 17.8 |
| Shoshone National Forest, WY | 72.2 | 53.2 | 73.5 | 77.2 | 77.6 | 40.1 | 23.2 | 35.4 | 32.8 | 26.1 |
| Big Horn National Forest, WY | 67.5 | 48.4 | 67.5 | 72.8 | 72.8 | 33.9 | 20.4 | 31 | 18.8 | 25.2 |
| Custer State Park, SD | 79.9 | 58.5 | 77.5 | 76.7 | 65.7 | 46.5 | 27.5 | 45 | 44 | 46.1 |
| Glacier National Park, MT | 70.8 | 50 | 70.3 | 80.8 | 64.9 | 40.8 | 26.6 | 39 | 40.5 | 38 |
| Wallowa-Whitman National Forest, OR | 65.6 | 45.6 | 64.5 | 69.2 | 69.3 | 31.2 | 22.8 | 26.7 | 21.6 | 22.1 |

Supplementary Table 6: PRISM data of vapor pressure deficit minimum and maximum

|  | Vapor pressure deficit minimum (hPa) | | | | | Vapor pressure deficit maximum (hPa) | | | | |
| --- | --- | --- | --- | --- | --- | --- | --- | --- | --- | --- |
| Site | 30 year normal | 2016 | Month sampled | Day sampled | Day before sampling | 30 year normal | 2016 | Month sampled | Day sampled | Day before sampling |
| Mt. Pinos, CA | 5.95 | 3.1 | 7.36 | 7.81 | 4.75 | 18.91 | 14.6 | 28.71 | 25.88 | 28.27 |
| Great Basin National Park, NV | 3.16 | 2.25 | 3.06 | 2.03 | 0.92 | 15.95 | 8.92 | 12.89 | 9.45 | 7.05 |
| Valles Calderas National Park, NM | 1.54 | 1 | 0.28 | 0.89 | 0.35 | 18.14 | 11.19 | 13.62 | 21.7 | 18.21 |
| Durango, CO | 2.66 | 1.8 | 1.65 | 4.47 | 4.48 | 20.25 | 10.01 | 13.89 | 19.13 | 20.47 |
| Rocky Mountain Biological Laboratory, CO | 2.09 | 1.11 | 1.56 | 0.99 | 0.82 | 14.07 | 9.73 | 15.18 | 11.51 | 16.36 |
| Rollins Pass, CO | 1.62 | 1.87 | 2.95 | 6.12 | 6.34 | 15.98 | 10.18 | 16.35 | 22.82 | 20.15 |
| Niwot Ridge (Forest Site), CO | 1.94 | 1.64 | 2.61 | 1.1 | 1.86 | 13.41 | 8.84 | 14.2 | 15.61 | 15.75 |
| Niwot Ridge (Treeline Site), CO | 2.2 | 1.61 | 2.71 | 0.79 | 1.87 | 10.33 | 7.71 | 12.39 | 13.02 | 13 |
| Rocky Mountain National Park, CO | 1.02 | 1.28 | 1.92 | 6.05 | 2.57 | 10.89 | 8.83 | 14.84 | 22.03 | 20.62 |
| Medicine-Bow National Forest, WY | 2.09 | 0.96 | 1.39 | 1.28 | 1.67 | 21.29 | 11.51 | 22.64 | 25.25 | 25.59 |
| Tie Siding, WY | 3.59 | 2.42 | 5.69 | 0.6 | 2.99 | 20.5 | 8.97 | 18.57 | 6.9 | 16.47 |
| Shoshone National Forest, WY | 3.32 | 1.57 | 2.54 | 2.67 | 3.7 | 19.34 | 10.79 | 21.9 | 25.71 | 29.73 |
| Big Horn National Forest, WY | 3.79 | 1.62 | 3.85 | 5.85 | 5.9 | 17.69 | 8.18 | 17.49 | 23.99 | 23.21 |
| Custer State Park, SD | 1.5 | 0.9 | 1.04 | 0.47 | 0.05 | 24.35 | 12.64 | 22.28 | 21.48 | 10.77 |
| Glacier National Park, MT | 2.54 | 1.26 | 1.78 | 0.9 | 1.22 | 19.51 | 8.32 | 16.33 | 27.05 | 6.83 |
| Wallowa-Whitman National Forest, OR | 4.54 | 1.5 | 4.06 | 7.61 | 8.79 | 16.74 | 6.37 | 15.71 | 20.72 | 20.14 |

Supplementary Table 7: Core ASVs. The table shows prevalence (as % of samples where the ASV is present) of ASVs with detection above 0.1% in each site (ASV short names are the same as in Supplementary Table 7).

| ASV | MP | GRBA | VCNP | DUR | RMBL | RP | NRFOR | NRTL | RMNP | MBNF | TS | SNF | BHNF | CSP | GNP | WWNF |
| --- | --- | --- | --- | --- | --- | --- | --- | --- | --- | --- | --- | --- | --- | --- | --- | --- |
| AAB1 |  |  | 70 | 60 | 95 | 60 | 75 |  | 65 |  | 75 | 70 |  | 65 | 75 |  |
| AAB2 |  |  | 60 | 70 | 95 | 70 | 80 | 80 | 75 |  | 70 |  | 85 | 65 | 50 |  |
| AAB3 |  |  |  |  | 95 | 55 | 65 |  | 60 |  | 65 |  |  |  |  |  |
| AAB4 |  |  |  |  | 80 | 65 | 75 | 75 | 70 |  | 55 |  | 75 |  | 80 | 75 |
| AAB5 |  |  |  |  |  |  | 60 |  | 55 |  |  |  |  |  |  |  |
| AAB6 |  |  |  |  |  |  |  | 75 |  |  |  |  |  |  |  | 75 |
| BURK1 |  |  |  |  |  |  |  |  |  |  |  |  |  | 65 |  |  |
| RHOD1 |  |  |  |  |  |  |  |  |  | 70 |  |  |  |  |  | 55 |
| AAB7 |  |  |  |  |  | 65 | 65 | 80 |  |  | 55 |  |  |  | 55 | 55 |
| ENT2 |  |  |  |  |  |  |  |  |  |  |  |  |  |  |  |  |
| AAB8 |  |  |  |  |  |  |  |  |  |  |  |  |  |  |  |  |
| COM1 |  |  |  |  |  |  |  |  |  |  |  |  |  |  |  |  |
| COM2 |  | 50 |  |  |  |  |  |  |  |  |  |  |  |  |  |  |
| BURK2 |  |  |  |  |  |  |  |  |  |  |  |  |  |  |  |  |
| ENT1 |  |  |  |  |  |  |  |  |  |  |  |  |  |  |  |  |
| COM3 |  |  |  |  |  |  | 55 |  |  |  |  | 55 |  | 65 |  | 55 |
| BLA1 |  |  |  |  |  |  |  |  |  |  |  |  |  |  |  |  |
| FLAV1 | 70 |  |  |  |  |  |  |  |  |  |  |  |  |  |  |  |
| AAB9 | 70 |  | 65 |  |  |  |  |  |  |  |  |  |  |  |  |  |
| COM4 | 65 |  |  |  |  |  |  |  |  |  |  |  |  |  |  |  |

Supplementary Table 8: Taxonomic identity of the overall top 20 most relatively abundant ASVs

| **ASV short name** | Phylum | Order | Family | Genus |
| --- | --- | --- | --- | --- |
| AAB1 | Alphaproteobacteria | Rhodospirillales | Acetobacteraceae |  |
| AAB2 | Alphaproteobacteria | Rhodospirillales | Acetobacteraceae | Acidiphilium |
| AAB3 | Alphaproteobacteria | Rhodospirillales | Acetobacteraceae |  |
| AAB4 | Alphaproteobacteria | Rhodospirillales | Acetobacteraceae |  |
| AAB5 | Alphaproteobacteria | Rhodospirillales | Acetobacteraceae |  |
| AAB6 | Alphaproteobacteria | Rhodospirillales | Acetobacteraceae |  |
| BURK1 | Betaproteobacteria | Burkholderiales | Burkholderiaceae | Burkholderia |
| RHOD1 | Alphaproteobacteria | Rhodobacterales | Rhodobacteraceae | Paracoccus |
| AAB7 | Alphaproteobacteria | Rhodospirillales | Acetobacteraceae |  |
| ENT2 | Gammaproteobacteria | Enterobacteriales | Enterobacteriaceae |  |
| AAB8 | Alphaproteobacteria | Rhodospirillales | Acetobacteraceae |  |
| COM1 | Betaproteobacteria | Burkholderiales | Comamonadaceae | Delftia |
| COM2 | Betaproteobacteria | Burkholderiales | Comamonadaceae | Diaphorobacter |
| BURK2 | Betaproteobacteria | Burkholderiales | Burkholderiaceae | Burkholderia |
| ENT1 | Gammaproteobacteria | Enterobacteriales | Enterobacteriaceae | Pantoea |
| COM3 | Betaproteobacteria | Burkholderiales | Comamonadaceae |  |
| BLA1 | Bacteroidetes | Flavobacteriales | Blattabacteriaceae | Candidatus Uzinura |
| FLAV1 | Bacteroidetes | Flavobacteriales | Flavobacteriaceae | Cloacibacterium |
| AAB9 | Alphaproteobacteria | Rhodospirillales | Acetobacteraceae |  |
| COM4 | Betaproteobacteria | Burkholderiales | Comamonadaceae |  |

Supplementary Table 10: Shannon Diversity between host species within sites

| Site | Host Species | Mean Diversity | SD | n | Significance |
| --- | --- | --- | --- | --- | --- |
| GNP | PICO | 1.78 | 0.734 | 24 | p = 0.008057* |
| GNP | PIFL | 2.39 | 0.95 | 18 |  |
| GNP | PSME | 3.25 | 0.898 | 5 |  |
| WWNF | ABCO | 2.63 | 0.733 | 15 | p = 0.749344 |
| WWNF | PIFL | 2.61 | 0.987 | 32 |  |
| BHNF | ABCO | 2.95 | 0.827 | 9 | p = 0.529767 |
| BHNF | PICO | 2.7 | 0.618 | 21 |  |
| BHNF | PIFL | 2.51 | 0.8 | 17 |  |
| CSP | PIFL | 1.67 | 0.714 | 34 | p = 0.000071* |
| CSP | PIGL | 2.92 | 0.804 | 13 |  |
| SNF | PIEN | 2.26 | 0.697 | 14 | p = 0.495854 |
| SNF | PIFL | 2.56 | 0.924 | 23 |  |
| SNF | PSME | 2.29 | 1.13 | 10 |  |
| TS | PICO | 2.67 | 0.851 | 20 | p = 0.662748 |
| TS | PIFL | 2.79 | 0.849 | 20 |  |
| TS | PIPO | 2.95 | 1.26 | 7 |  |
| MBNF | PIFL | 2.57 | 1.02 | 25 | p = 0.493463 |
| MBNF | PIPO | 2.11 | 0.82 | 14 |  |
| MBNF | PSME | 2.18 | 0.788 | 8 |  |
| RMNP | ABLA | 2.08 | 0.49 | 18 | p = 0.004817* |
| RMNP | PIEN | 2.26 | 0.435 | 12 |  |
| RMNP | PIFL | 2.74 | 0.604 | 17 |  |
| NR_TL | ABLA | 2.65 | 0.736 | 16 | p = 0.985007 |
| NR_TL | PIEN | 2.72 | 0.706 | 17 |  |
| NR_TL | PIFL | 2.58 | 0.805 | 14 |  |
| RP | PICO | 2.39 | 0.884 | 20 | p = 0.101590 |
| RP | PIEN | 2.82 | 1.31 | 9 |  |
| RP | PIFL | 2.8 | 0.595 | 18 |  |
| GRBA | PIEN | 2.0 | 0.555 | 16 | p = 0.004628 |
| GRBA | PIFL | 2.93 | 0.961 | 13 |  |
| GRBA | PILO | 2.93 | 1.09 | 18 |  |
| RMBL | PIFL | 2.49 | 0.46 | 27 | p = 0.458460 |
| RMBL | PSME | 2.29 | 0.602 | 18 |  |
| DUR | ABCO | 2.66 | 0.996 | 7 | p = 0.280624 |
| DUR | PIFL | 2.62 | 0.814 | 17 |  |
| DUR | PSME | 2.17 | 0.626 | 10 |  |
| VCNP | PIEN | 2.51 | 1.09 | 10 | p = 0.101504 |
| VCNP | PIFL | 2.06 | 0.763 | 37 |  |

Supplementary Figure S1: Heatmap of the top 20 ASVs across sites and species. (ASV short names are the same as in Supplementary Table 7.)

Supplementary Figure S2: Shannon diversity by species for each site
